# The dual nature of constraints on foreign worker participation in sports and physical activity in South Korea during COVID-19

**DOI:** 10.3389/fspor.2023.1181414

**Published:** 2023-05-22

**Authors:** Dongkyu Na, Mihwa Park

**Affiliations:** Center for International Studies, Inha University, Incheon, South Korea

**Keywords:** constraint, foreign worker, E-9 visa, Foucault, COVID-19

## Abstract

This study provides a different understanding of the constraints imposed by the pandemic and the official and unofficial restrictions that accompanied it. It is an empirical effort demonstrating that the pandemic's effects are not purely negative, but rather, also helped to produce positive and productive practices that draw upon both the inhibiting and enabling features of the constraints it triggered. Engaging with “productive power” in Foucault by considering constraints as practices that both inhibit and enable, the empirical goal of this paper is to explore how pandemic-related constraints on sports and physical activity prohibit foreign worker participation in sports and physical activity. It also examines how the constraints encourage them to pursue an active life in new and unique ways. To achieve this goal, the paper examines the South Korean context, particularly unskilled foreign workers with E-9 visas for non-professional employment in the fishing, farming, and manufacturing industries and their involvement in sports and physical activity during the COVID-19 pandemic. The findings address three “inhibitors” that specifically prevented foreign workers from getting actively involved, then demonstrate that explicit restrictions on sports and physical activity can be transformed into four “enablers” that encouraged foreign workers to participate. The conclusion offers critical reflections on Foucault's “ethical subject,” followed by the limitations and implications of the study.

## 1. Introduction

Over the past three years, the COVID-19 pandemic prompted a seemingly endless number of lockdowns and restriction policies that prevented people from taking part in outdoor activity, sports, and physical activity in general. Physical activity and sports program providers such as schools, community centers, gyms, and fitness centers were forcefully shut down and social distancing discouraged people from getting together for any form of outdoor activity. A consequence of such movement constraints was that it had a disproportionate effect on minority groups, who had already faced barriers preventing their participation in social and cultural programs, including sports and physical activity (1). Colley and Watt (2) write that the COVID-19 pandemic seriously impacted youth participation in physical activity in Canada, causing the number of youth participating at levels meeting Canadian governmental recommendations to drop from 50.8% in fall 2018 to 37.2% just two years later. It was reported in the 2022 ParticipACTION Report Card on Physical Activity for Children and Youth that only 35% of immigrant youth met official recommendations for physical activity of moderate to vigorous intensity (MVPA) during the pandemic, compared to 56% before. In addition, the pandemic increased constraints on minority group participation in sports in the United Kingdom, disproportionately impacting minority groups so much that between May 2020 and May 2021, only 48% of Asian adults (excluding Chinese) met the UK's Chief Medical Officer's recommendations for leading an active life, compared to 63% of white Brits during the same period (3). Similarly, South Korea's 2020 White Sport Report (4) presented similar findings: overall sports and physical activity dropped due to nationwide restrictions, with once-weekly participation in athletic activities between 2019 and 2020 falling from 66.6% to 60.1%, and more than twice-weekly activity falling from 52.2% to 47.0%.

It is important to note that in the above literature, the COVID-19 pandemic and all relevant policy measures and environments are treated as “constraints” upon the actions of individuals, and focus on their subsequent “negative” effects on individual participation in sports and physical activity. However, these constraints should not simply be seen as negative practices that completely prevented physical activity, but rather, new realities that allowed for the discovery of new ways to enjoy sports and physical activity belonging to the era of the so-called contract-free society (5), which prohibits people's direct contact with others. For example, new methods for enjoying sports and physical activity online, and complementary digital platforms which emerged during this time, are worthy alternatives to the pre-pandemic, traditional way of leading an active life. While the pandemic has intensified and increased barriers to sports and physical activity, it has also provided an opportunity for people, as argued by Hayton (7), to think outside the box and “apply solutions to the constraints of the pandemic” (p. 13). Thibault et al. (8) also point out that while people experienced unprecedented outdoor restrictions, it also pushed us to consider and newly strategize about how we might participate in sports in different settings. This is reflected by the popularity of so-called “light sports” and the blending of “traditional and innovative sports settings” (p. 468) that would help recover the large drops in participation in sports and physical activity seen during the pandemic (8).

Keeping with that, this study provides a different understanding of the constraints imposed by the pandemic and the official and unofficial restrictions that accompanied it. It is an empirical effort which demonstrates that the effects of the pandemic are not purely negative, but rather, also helped to produce positive and productive practices that draw upon both the inhibiting and enabling features of the constraints it triggered. That is, in this paper, pandemic-induced constraints should not only be considered to have prohibited action, but to have also “inhibited” and “enabled” it. For example, Golob and Giles (9) approach leisure constraints on immigrants in Canada (which are treated as “both inhibiting and enabling individuals’ actions” in their research) from a Foucauldian perspective. In Foucault's (10) view, power is not just negative and restrictive, but also positive and relational. As such, constraints can be seen as Foucault's understanding of power, which is “productive, and therefore, never wholly limiting” (11). Thus, this paper carries the theoretical aim to fully engage with “productive power” in Foucault's (10) theory by considering constraints as practices that both inhibit and enable. The empirical goal of this paper is to explore how pandemic-related constraints on sports and physical activity not only prohibited foreign worker participation in sports and physical activity, but also encouraged them to pursue an active life in new and unique ways. To achieve this goal, the paper examines the South Korean context, particularly unskilled foreign workers with E-9 visas for non-professional employment in the fishing, farming, and manufacturing industries and their involvement in sports and physical activity during the COVID-19 pandemic.

The paper first provides a brief overview of foreign workers holding E-9 visas in South Korea, and discusses how their participation in sports and physical activity was constrained by social and policy settings before and during the pandemic. It then presents a theoretical framework, based on Foucault's concept of power, that shows constraints are practices that both inhibit and enable action in the pandemic context. It is followed by details about the study's design, including the processes of data collection and analysis; and the findings, which reveal a range of difficulties and challenges that foreign workers faced when getting involved in sports and physical activity during the pandemic, and how these constraints were understood as practices that either inhibited or enabled their participation during the pandemic. Lastly, the conclusion offers critical reflections on how to be an “ethical subject,” represented by Foucault's (12) technologies of the self, followed by the limitations and implications of the study.

## 2. Constraints on E-9 holders in South Korea

The 1980s marked rapid change within Korea: along with increasing economic development, the tendency for Koreans to avoid the so-called 3D (dirty, difficult, and dangerous) industries worsened, leading to demand for foreign workers to fill this labor gap (13). Developing countries encouraged their citizens to go abroad, eventually leading to the gradual growth of the foreign population in South Korea. On the policy side, the Korean government implemented the Vocational Training System policy in 1993, which invited foreign workers to come as trainees to improve their job skills and work in small to medium-sized labor-intensive industries (14). However, this was an employer-centric policy that only focused on labor supply for the unpopular industrial sector, which often subjected workers to chronic issues including illegal or unsafe employment, wage abuse and theft, and even physical abuse in some cases (14).

In reaction, the Employment Permit System (EPS) was introduced in 2004 with the aim of helping small- to medium-scale businesses in the agricultural, fishery, construction, and manufacturing industries solve their labor shortages and to also strengthen the legal employment of foreigners in Korea through “transparency of the foreign selection and introduction process, and prevention of corruption practices and anomalies in the sending process” (15). Through the EPS, foreign workers entering South Korea are given the E-9 non-professional work visa which makes them eligible to work in labor-intensive sectors. The main bulk of foreign workers in Korea are from 16 developing countries in Asia: Bangladesh, China, Cambodia, Indonesia, Kyrgyzstan, Laos, Mongolia, Myanmar, Nepal, Pakistan, Philippines, Sri Lanka, Thailand, Timor-Leste, Uzbekistan, and Vietnam (16). According to the Ministry of Justice's Korea Immigration Service (17), there were 217,729 unskilled foreign workers with E-9 visas as of December 2021. As shown in Table 1 below, Cambodia, Nepal, Vietnam, and Thailand are the top sending countries. Table 2 shows the number of foreign workers by province in South Korea (18). The majority live in Gyeonggi province, with its two major cities Hwaseong and Gimpo being home to the most foreign workers, at 17,253 and 9,401, respectively.

**Table 1:** Number of foreign workers by country.

Country	Foreign workers	Country	Foreign workers
Cambodia	33,261	Uzbekistan	9,482
Nepal	28,285	Bangladesh	7,253
Vietnam	27,651	Mongolia	4,326
Thailand	22,478	Pakistan	2,317
Indonesia	20,830	Timor-Leste	2,221
Myanmar	20,328	China	1,633
Philippines	19,390	Kyrgyzstan	684
Sri Lanka	16,508	Laos	331
Total	216,978		

**Table 2:** Number of foreign workers by province in Korea.

Province	Foreign workers	Province	Foreign workers
Gyeonggi	88,919	Daegu	5,069
South Gyeongsang	26,455	Gangwon	3,843
South Chungcheong	19,703	Ulsan	3,782
North Gyeongsang	15,602	Gwangju	3,512
South Jeolla	14,055	Jeju	3,498
North Chungcheong	13,743	Seoul	2,662
Incheon	10,877	Sejong	1,231
North Jeolla	8,420	Daejeon	808
Busan	6,183		
Total	1,089,791		

Although foreign workers are only able to work for three years on the E-9 visa, they are eligible for a maximum of four years and ten months (58 months) through a one year and 10-month extension that their employer must apply for (18). After the end of the 58-month visa period, they must return to their home country and apply there for another visa if they choose to return. However, the approval of a second visa is not guaranteed and has particular requirements, including the conditions that foreign workers who apply again must work in the same industry, they must be re-employed by their previous employer (from their previous visa) and work for them for at least one year, and that their previous employer must strongly intend to re-hire the applicant (19). Under the EPS, foreign workers with E-9 visas are unable to invite their spouse and children to come live with them. This is a policy effort to prevent the settlement of non-professional foreigners in Korea, which allows the government to avoid taking responsibility for the social costs generated from their settlement (20). In this respect, it can be seen that foreign workers with E-9 visas experience institutional exclusion from Korean civic society, as their access to visa extensions is fundamentally controlled by employers and they are treated as aliens or “others” during their time in Korea. Furthermore, it is also important to note that non-professional foreign workers with E-9 visas experience restrictions on changing workplaces or industries during their period of stay. Such constraints include being unable to change the first workplace and industry type written in their original work contract without a legitimate reason, and only being able to change employers or industry a maximum of three times within three years if they can prove that their current employer: (1) asked to terminate the contract, (2) closed their business, (3) violated the employment contract, (4) or that the foreign worker got in trouble for retaining their current jobs in the way that they lost their legal status, committed a crime, or run away from their workplace (18).

There are 40 support centers for foreign residents in South Korea, shown in Table 3. These centers provide a range of cultural programs that help aid the social integration of foreigners into Korea, including cultural events, providing a platform to meet and interact with local residents, and various sports and leisure programs (21). However, it is rather difficult for non-professional foreign workers to access the support centers because they usually live at accommodations provided by their employer, and their workplaces are often located far away in more suburban or rural areas due to the nature of their industry (22). In addition, foreign workers often show a preference for working overtime on weekdays or the weekend to receive more wages, rather than spend time participating in cultural or sport programs (22, 23). Other constraints that inhibit foreign workers' participation in sports and physical activity can be a lack of spare time, financial concerns, and unfamiliarity with Korean sports culture (24). It is also reported that foreign workers are discouraged from participating in the Korean sports community because they are consciously treated as “less-developed others” in the world, due to them coming from low- or middle-income countries in Asia (25).

**Table 3:** Number of foreign support centers by province.

Province	Number of foreign support centers	Province	Number of foreign support center
Gyeonggi	11	Daegu	1
South Gyeongsang	5	Gangwon	1
South Chungcheong	3	Ulsan	1
North Gyeongsang	3	Gwangju	3
South Jeolla	3	Jeju	1
North Chungcheong	1	Seoul	2
Incheon	2	Daejeon	1
North Jeolla	2		
Total	40		

In particular, the pandemic exacerbated constraints on foreign worker participation in sports, physical, and leisure activities. On the policy level, the principal factor that discouraged foreign residents from engaging was actually an administrative order requiring all foreign workers in South Korea to get tested for COVID-19. While it was stated this order was made for the purpose of preventing viral spread, it served to label and treat foreign workers as people “suspected” of infection (26). This was a race-based discriminatory policy that clearly distinguished foreign workers from Korean citizens, as it carried the inherent message that foreign workers were responsible for spreading the virus (27). Such a government approach led to broader discrimination against foreign workers in the public sphere, as they were seen as suspected or likely cases of infection in public places and facilities like parks, hospitals, restaurants, and gyms (28). An additional policy was implemented that excluded foreign workers without national health insurance from getting face masks from the government, and thus foreign workers were further restrained from going outside (28).

The pandemic led to lower incomes and the loss of livelihood for many, which can be seen as another constraint on foreign worker involvement in sports and physical activity. In fact, in reaction to a decline in workload, some businesses chose to reduce the working hours of their foreign employees or lay them off entirely (14). This naturally led to income reduction and functioned to inhibit sports participation. Some workers also reported not getting paid when they were quarantined after getting infected, and some employers prohibited foreign workers from going out under the pretext of protecting their health (29). Framing such actions as being necessary to prevent virus spread, foreign workers were discriminated against in various ways: their movement was restricted to their work dormitories, their actions were monitored by their employers, and their participation in cultural programs at the foreign support centers was prohibited (14). In the initial stage of the COVID-19, such restrictions only targeted foreign workers, but were not applied to Korean workers. Furthermore, real-time news and information about the pandemic in South Korea were only provided in Korean and English, although the policy restrictions and social distancing recommendations themselves were translated into the 16 languages spoken by the largest foreign worker populations (28). That is, without the provision of critical news and updates in their native languages, foreign workers could only access materials on restrictions and other preventative measures, making them reluctant to go outside and expose themselves to potential infection (30). In this respect, this can be another constraint that hindered foreign workers from participating in sports in South Korea during the COVID-19 pandemic.

In general, literature on constraints caused by the pandemic mainly focused on its “negative” aspects which produced “suppressive,” “restrictive,” and “intolerant” practices discouraging foreign workers from sports participation in any way. In contrast, this paper attempts to shed light on some “positive” constraints that promoted “productive,” “alternative,” and “new” practices and actions enabling their participation during the pandemic.

## 3. Constraints on sports and physical activity participation, COVID-19, and Foucault

In Foucault's (31) view, power is relational and productive rather than related to negative modes of power involving “the use of any repressive and violent means” (p. 242). Power does not exist in a vacuum, but in relations, and social reality in Foucault is understood as “the product of power relations” (32), contingent upon the effects of everyday relations between individuals and institutions. In this form of power, it is wrong to understand the way in which individuals are directed by “totalizing forces without possibilities for resistance or change” (33), but to see the room for individuals with “the possibility of action upon the others (which is coextensive with every social relationship)” (34). Following that, the actions of athletes and participants in sporting practice are not unilaterally restricted by constraints of games such as rules, requirements, and legitimate skills (11). Rather, such constraints of the games also produce possibilities of enabling individuals to accept, adjust, and/or change their actions that fit the rules of a particular sporting practice. In this vein, following Foucault's understanding of positive and relational power Debra Shogan (35) regards power as “constraints on action” and embraces the way in which these constraints “either inhibit or enable action” (p. 3). It is plausible to, argued by Shogan (35), understand rules of sports as constraints that “prescribe certain actions, proscribe other actions, and describe boundaries or contexts within which these actions make sense” (pp. 4–5). These constraints produce particular actions by limiting the actions of individuals in the rules of sports sticking to a single way or the combination of the three ways, prescriptive, proscriptive, and descriptive rules (35). At the same time, they also make possibilities for athletes and participants by enabling them to change, remove, and/or adopt their actions pertinent to rules of a particular sporting practice (11).

Following Foucault, Shogan's (11, 35) these approaches to constraints provide theoretical rationale for follow-up studies concerning the dualism of constraint to both be “inhibiting” and “enabling” actions, particularly in leisure, sport, and a range of physical activities. For example, Giles (36) draws on the understanding of “power as constraints on action that are both enabling and inhibiting” (p. 15) to find a new, alternative approach to the research involving Dene women, menstrual traditions, and participation in physical practice such as different forms of sports, physical activity, recreation, and Dene's traditional games in the Dehcho (Mackenzie) region of the Northwest Territories (NWT) in Canada, through using Foucault. In Giles' (36) findings, Foucault's (10) understanding of constraints to be positive allows to see the inhibition of Dene women's participation in physical practice during menstruation as a way of positively enabling discourses, rather than absolutely inhibiting discourses about Dene women and their physical practice which has been ignored in the exclusive use of an Indigenous research framework. By taking up Foucauldian understanding of power as constraints on action that “simultaneously enable and inhibit action allow researchers to explore” (p. 313) how individual's participation in leisure activities and its experiences are constructed as productive, positive, and active, Golob and Giles (9) direct their analysis on constraints on immigrants' leisure in Canada. They disclosed the way in which discourses of Canadian multicultural citizenship produce constraints that both prohibit and allow for immigrants' leisure pursuit, thereby convincing the useful application of a Foucauldian approach to leisure and sports studies. Mills et al. (37) use Foucault's disciplinary framework, particularly focusing on discourse, power, and the subject, to examine the first author's experience as a former runner and “inherent constraints” (p. 5) on athlete's high-performance achievement, produced by coaching “truths” trenched in everyday bioscientific and post-positivist knowledge. While the Mills et al.'s (37) were not directly meant the dualism of constraints that are both inhibiting and enabling action, the “inherent constraints” in the analysis of the study following Foucault's power and its positive/relational operation can be seen that inherent constraints are not just “problematic within a high-performance sports context” (p. 5), but they also produce a possibility to break the ideological coaching truths.

This is reminiscent of Shogan's (11) cooperative roles of proscriptive and prescriptive constraints. While constraints proscribe particular actions of individuals, they also “make other actions possible” (p. 29) by prescribing the ways in which individuals' actions are legitimately allowed in “an unchaotic environment” (p. 29). Considering the COVID-19 and restrictions on outdoor activity, they are explicitly seen as proscriptive constraints that prohibit individuals from participating in sports and to physical activity. At the same time, the constraints can also produce a “safe” sporting practice in the way that sports participation is prescribed in an unchaotic setting. It is also noteworthy taking a closer look at Mills et al.'s (37) hope from their study, especially reinforced by Foucault's theoretical framework. That is, Mills et al.' s (37) theoretical hope is to challenge authoritative coaching practice that produces proscriptive constraints, referred to as problematic, and prescriptive constraints, represented by bioscientific truths. At this moment, this approach to constraints can be linked to Foucault's (38) technologies of the self, which “permit individuals to effect by their own means … so as to transform themselves in order to attain a certain state of happiness, purity, wisdom, perfection, or immortality” (p. 18). For Foucault, subject is a form that can be adjusted depending on a variety of contexts, and thus the technologies of the self is the way that individuals “transform” their subjects fitting to a given practice through the process of “self-reflection and self-examination” (33, p. 143), as well as critical thinking of “normal” things. In this respect, the application of a Foucauldian approach to constraints can be useful for the examination of how a range of constraints during the COVID-19 could be both inhibiting and enabling foreign workers' participation in sports and physical activity. Our theoretically-informed analysis of the study is an effort to fill in the gaps in the literature using Foucault's understanding of constraints in leisure, sports, and physical activity, which has had little follow-up empirical research since Golob and Giles's (9) work. Furthermore, shown in Giles' (36) and Mills et al.'s (37) research, the use of a Foucauldian approach to foreign workers' experience in the constraints of the COVID-19 may enable us to identify subjects of foreign workers in the working of the technologies of the self through the way in which they reflect on the reasons and importance of doing sports and physical activity even during the pandemic.

## 4. Study design

This research project was based on the first author's experience getting involved in sports and physical activity, especially regarding his struggles with restrictions and outdoor activity constraints in Turkey, Canada, and South Korea during the pandemic. Mills (39) remarked, “the most admirable thinkers within the scholarly community you have chosen to join do not split their work from their lives” (p. 195). During the first author's time in Turkey and Canada during 2020 and 2021, lockdowns, the closure of public facilities, and outright social gathering bans prevented him from participating in outdoor activity. Importantly, the first author's identity as a foreign national from Asia made him even more reluctant to go out and enter public spaces when he realized that local residents seemed especially wary of individuals who looked Asian. He felt minimally motivated by the new and alternative methods of sports that arose during this time, including online programs, because they were mostly provided for local residents in the local language, rather than for foreigners. Nevertheless, the first author's pursuit of leading an active life and his obsessive love for the outdoors meant that he did not give up by any means, instead choosing to do activities, for example, jogging, running, and hiking alone to comply with distancing restrictions. He was unmotivated by online programs and opted to find alternatives that allowed him to stay active outside, and in-person if possible. It eventually led to a new love for tennis, which was relatively safe from social distancing restrictions. This indicates that pandemic-related constraints are not purely negative and prohibitive, manifested by the constraints that prevented the first author's traditional approach to sports participation, but also allowed for his transformation into an active subject in sports participation. In other words, the first author's experience in choosing not to pursue more conventional methods of sports participation pushed him to look at the positive things that could come from the constraints, including new and innovative ways to live actively.

Focusing on the features of constraints that both inhibit and enable (9), this study concerns unskilled foreign workers holding the E-9 work visa in South Korea and their participation in sports and physical activity during the COVID-19 pandemic. To pursue the goal of this study, we conducted semi-structured interviews. During the pandemic, outdoor movement and direct contact with other people were strictly prohibited, and thus the authors needed to wait until pandemic-related restrictions were loosened by the Korean government to conduct interviews with the study participants in person. Furthermore, this study was an initial step to understand the dual features of constraints that both serve as inhibitors and enablers of foreign workers' involvement in sports, leisure, and physical activity in South Korea. The role of semi-structured interviews is to elicit “lived experience while also addressing theoretically driven” questions (40). Therefore, we employed the semi-structured interview following a Foucauldian approach to constraints, to lay the foundation for future studies utilizing different research methods. It is also worth focusing on Galletta's (40) emphasis on the importance of “reciprocity” and “reflexivity” in conducting semi-structured interviews. To achieve considerable reciprocity with interview participants and researcher reflexivity, it helps to share the researcher's experiences and their connection to the topic (40). In fact, Weninger and Dallaire (41) used semi-structured interviews to explore how female barrel racers in male-dominated sports in Western Canada differentiate themselves from other rodeo men and women, thus uncovering their unique interactions with their horses as a key difference. With emphasis on the understanding of a specific context relied upon the research setting, the principal author Desirea Weninger shared her autobiography and its relationship with the study topic. As a “skilled” foreign worker and doctoral student, the first author's experience in Canada and Turkey during the pandemic would not be the same as that of the study participants as “unskilled” foreign workers in this study, who are usually considered to be marginalized groups in South Korea (30). Similarly, before the pandemic, the first author had been aware of his identity as a minority and “outsider” in Canada and Turkey, and this self-categorization was felt more greatly under the seemingly tendency of citizen-centered policy measures and social environment during the pandemic. Nevertheless, the first author expected that his experience of the pandemic constraints that enabled him to transform into an active subject in his own life, rather than being a restricted and subjugated one, would help him develop more comprehensive understanding of foreign workers in South Korea beyond more traditional or conventional views and perspectives. It is reminiscent of Lucas and Jeanes' (42) ethnographic research on the power relations that emerge from the privileged knowledge of Northern volunteers and the localized voices of Southern recipients. Based on the experience of the first author as a Northern volunteer in the Solomon Islands, the study reveals the ways in which local agents were not simply passive recipients of Northern-driven initiatives, but rather, reject the Northern knowledge and expertise.

The study was approached by the Inha University Institutional Review Board. Recruitment of interviewees sought to purposefully recruit unskilled E-9 visa holders who had actively participated in sports and physical activity in Hwaseong and Gimpo in Gyeonggi province, South Korea by using a purposeful, criteria-based sampling strategy to “yield the most relevant and plentiful data” (43). As shown in Table 2, the majority (84,804, 38.9%) reside in Gyeonggi province, and the cities Hwaseong and Gimpo have the biggest populations of unskilled foreign workers with E-9 visas in the province: 17,253 (out of 84,804, 20.3%) and 9,401 (out of 84,804, 11%), respectively (17). Thus, the second author first contacted the two cities' representative foreign support centers, Hwaseong Immigrants Community Service Center and Gimpo Foreign Resident Support Center, which aim to contribute to the social integration of foreigners by offering a variety of administrative services, educational courses, and social, cultural, and sports programs (44). The second author had previously conducted a series of research projects on migrant workers through the two centers, and had already established a strong rapport with them “by conveying empathy and understanding without judgment” as Patton (45) suggested. Thus, she already had ample knowledge about the centers' programs and the foreign residents they served. For the study, she identified that the two centers possessed sufficient numbers of foreign workers who (1) were actively involved in sports and physical activity, either provided by the centers or elsewhere, and (2) those with experience participating during the pandemic, particularly from the start of the pandemic in March 2020 to the loosening up of restrictions in April 2022. In recruiting participants for this study, authors explained our interest in comprehensively understanding different forms of constraints on foreign workers' participation in sports during the pandemic with hope to contribute to the improvement of foreign workers' integration into Korean society. Informed consent was obtained from all participants who were aware of their right for anonymity and confidentiality in the study. It was also explained that the collected data would be remained in the authors' password-protected personal laptops. Accordingly, 10 foreign workers, as participants from Nepal, Indonesia, Vietnam, Mongolia, Thailand, and Cambodia, were finally recruited, all with different experiences during the pandemic, as shown in Table 4. To protect the identity of the participants, the participant names were pseudonyms based on popular names in their home countries.

**Table 4:** Demographics of the study participants.

	Participant (pseudonyms)	Age	Country	Residence	Sport/PA
A	Amir	28	Nepal	Hwaseong	Taekwondo
B	Ram	26	Nepal	Hwaseong	Taekwondo
C	Farrel	37	Indonesia	Hwaseong	Table tennis
D	Binh	39	Vietnam	Hwaseong	Badminton
E	Khaliun	34	Mongolia	Hwaseong	Football
F	Hung	40	Vietnam	Gimpo	Football
G	Ngai	30	Vietnam	Gimpo	Football
H	Chakkrit	33	Thailand	Gimpo	Football
I	Samnang	34	Cambodia	Gimpo	Taekwondo
J	Oudom	30	Cambodia	Gimpo	Taekwondo

During the undertaking of this study, the semi-structured interview protocol was designed based on the Foucauldian understanding of constraints and its applied literature on sports, physical activity, and leisure constraints, as well as literature on outdoor activity during the COVID-19 pandemic. The interviews were guided by three main questions. (1) What were the difficulties and challenges that foreign workers faced when getting involved in sports and physical activity during the pandemic? (2) How did they understand and deal with constraints on participation in sport and physical activity, imposed by pandemic-related restrictions and environments? (3) And how were the experiences of foreign workers under these constraints, which motivated them to actively participate in sports and physical activity? Focusing on these main questions, 10 interviews were conducted in the locus of the two centers, and interpreters sat with the foreign workers the entire time. Having interpreters present allowed the authors to conduct interviews with the participants in their native languages (Nepalese, Indonesian, Vietnamese, Mongolian, Thai, and Cambodian). Each interview was recorded and lasted from 45 to 70 min.

**Figure 1:**
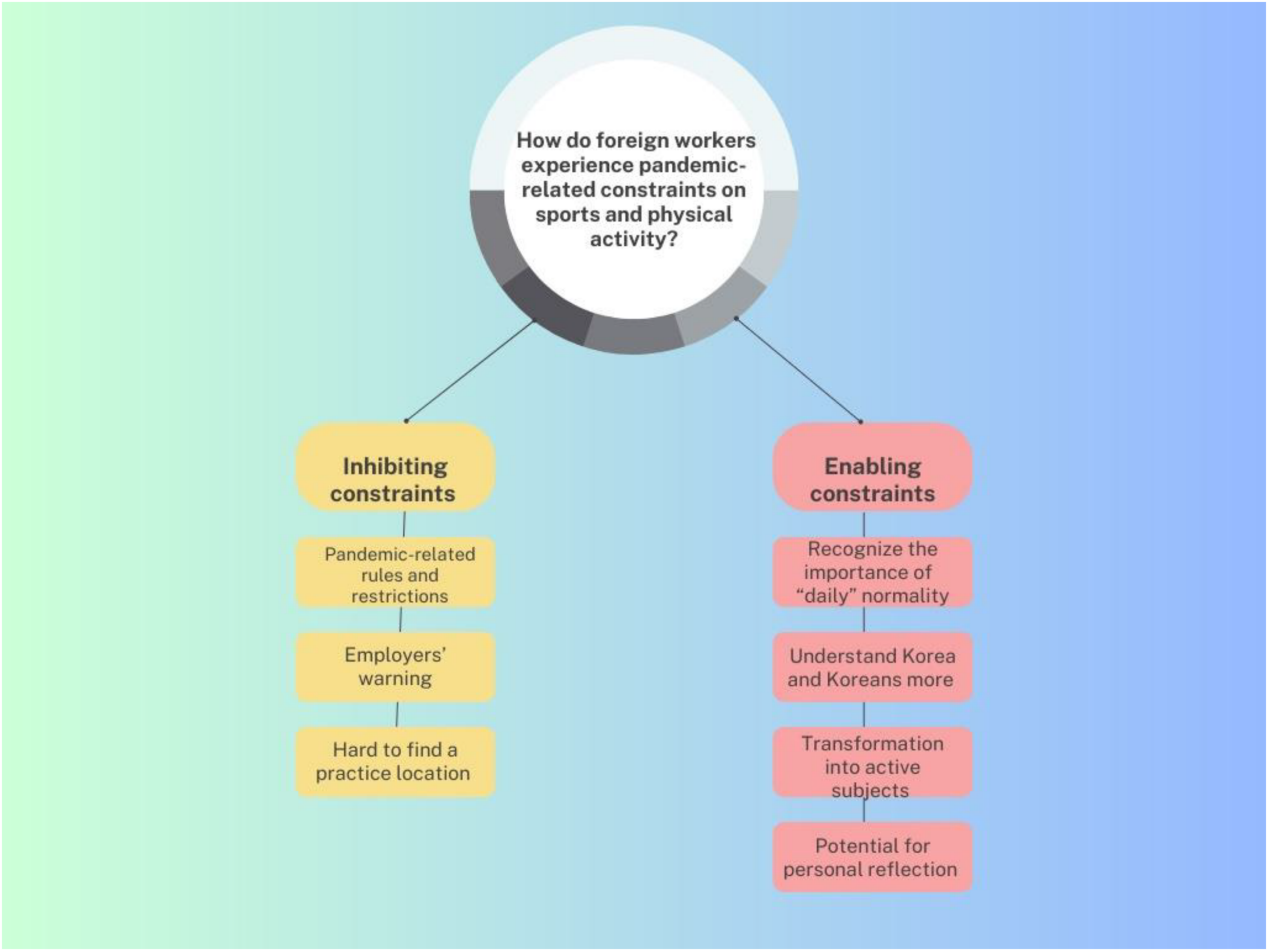
Categorization of major themes and subthemes.

To analyze the data gathered from the interviews, a hybrid approach of thematic analysis was selected in the way in which deductive approaches provided an initial sound of major themes driven by the theoretical framework following Foucault, and an inductive process for the subthemes and codes was carried out to clearly represent the data. In this study, the hybrid thematic data analysis involved three stages as described by Miles and Huberman (46): (1) data reduction. (2) data display, and (3) conclusion drawing and verification. The data reduction stage started with data documentation. At an early stage of the data analysis, data was organized by the participants. Then all interviews were first transcribed verbatim through Naver Clova Note, a software program developed by a Korean search portal and online platform, yielding 143 pages of data. After organizing and transcribing the data, the stage of data display involved exploring the data and generating the codes. Both authors individually read data transcriptions several times to get a sense of the data. While reading the transcriptions, the authors made notes, and highlighted important passages, phrases, and ideas. The further process of the data analysis engaged in deductively coding the data according to a coding frame developed by the authors. In this stage, the authors coded the raw data separately. When a new set of codes emerged inductively, the coding frame was updated. And then the authors reread through the raw data and iterated on the codes and labelled according to the new coding frame. This process was used to establish categories, which were then conceptualized into broad themes and minor themes in alignment with the research questions after further discussion among the authors. This stage was finalized when the authors reached a saturation point in the way that new information about the list of the themes was hardly found. As a result, the analysis of the interviews led to aggregating the codes into two theoretical-based major themes, “inhibiting constraints” and “enabling constraints.” While the first theme concerned constraints that prevented foreign workers from participating in and continuing on with their favorite sports and physical activities during the pandemic, narratives related to constraints that allowed and encouraged their participation were sorted into the second theme seen in the Figure 1.

The final stage consisted of conclusion drawing and the verification of the data. Conclusion drawing was tentative and iterative in which the researchers read the transcription and then continued with carefully listening to each recorded interview and compared them with the generated transcription. During this stage, the narratives and explanatory texts were developed to associate the Foucauldian theoretical interpretation with the context, social background, and excerpts of the participants shown in the following section. To support or repute early conclusions that were drown, the authors initially worked with interpretating the parts and the whole of the individual interviews and then moved between the whole of the other participants' data based on their personal experiences. The processes for verification included spanning the cycle of data analysis to enhancing the rigor of the research process. Throughout the analysis process, the initial ideas emerged from the raw data through early coding independently by the researchers, as well as their discussions until they reached a consensus. In addition, data narratives and excerpts were labelled with the participant codes and pseudonyms were assigned to each participant to guard their confidentiality. Multiple strategies were also used to ensure the trustworthiness and the credibility of the research process: member checking and debriefing [e.g., (47)]. After conducting interviews, the authors asked the participants to review transcript of their interviews and check their accuracy. Both authors also served as peer debriefers for different aspects of the research.

## 5. Findings

In this section, we present our findings on foreign workers experiencing constraints on sports and physical activity participation during the pandemic. From the analysis, the three subthemes of (1) Pandemic-related rules and restrictions, (2) Employers' warnings, and (3) Hard to find a practice location were noted to fall under the first theme of Inhibiting constraints, whereas the second theme of Enabling constraints consisted of the four subthemes of (1) Recognize the importance of “daily” normality, (2) Understand Korea and Koreans more, (3) Transformation into active subjects, and (4) Potential for personal reflection. Therefore, we first discuss the three “inhibitors” that specifically prevented foreign workers from getting actively involved, then demonstrate that explicit restrictions on sports and physical activity can be transformed into the four “enablers” that encouraged foreign workers to participate.

### 5.1. Inhibiting constraints

#### 5.1.1. Pandemic-related rules and restrictions

Government policies restricting outdoor gatherings, outdoor events, and even outdoor movement, in addition to mandated lockdowns, completely prevented foreign workers from participating in sports and physical activity. COVID-19 was an unprecedented crisis for society, and there was no feasible way to prevent the spread of infection besides outrightly banning social gatherings.

*During the first stage of the pandemic, the [taekwondo] program was stopped for about six months. Later on, in lieu of in-person classes, our gwanjangnim [instructor] recorded his poomsae [defined pattern of defense-and-attack motions] and kicks and sent those videos to us. We would watch the videos and practice by ourselves. (Amir, taekwondo)*

Similar to the aforementioned taekwondo program, on-site sports and activity programs at the two support centers were unavailable to foreign workers during the pandemic. Furthermore, relevant measures and alternatives in line with COVID restrictions also seemed to inhibit participation.

*There was no one to cheer us on, and I felt uncomfortable every time I had to get my body temperature checked when I got on the field. I was only able to participate in football games with a valid vaccine certificate … It was inconvenient to go through all that and always have required documents on me. (Khaliun, football)*

Like Khaliun, other foreign workers also felt that pandemic-related rules were inconvenient as they had to always be careful to wear masks, keep vaccine certificates on hand, and undergo body temperature checks. Restrictions such as social distancing and participant limits in certain spaces also discouraged foreign participants from playing in games that were made spectator-less (friends, family, or acquaintances who they might generally spend their spare time with were not allowed to attend). In reaction to the restrictions, online programs and platforms encouraging sports and physical activity were greatly promoted and improved upon, but these alternative methods were not thoroughly inclusive of foreign workers (1). Additionally, the support center mentioned above did not hold any live Zoom or Microsoft Teams activities, only providing recorded videos for taekwondo program participants. Similarly, the Gimpo Center provided a real-time online program but only for taekwondo, and most importantly, participants showed little interest in it. That is, online programs emerged as a solution for navigating restrictions, but they can be seen as constraints in the way that they inhibited foreign residents' participation in sports and physical activity.

*We couldn't get together because of COVID-19, so we could only do it [taekwondo] online. But it didn't take long before I quit because it was a bit inconvenient to practice by myself. When I did taekwondo in person, I could practice moves with a partner, but that wasn't possible online. I missed a lot of sessions and just ended up not attending anymore at all. (Samnang, football)*

Taekwondo is a martial art which can be practiced in isolation, thus making it suitable for shifting to online settings, but that wasn't the case for team sports. Similarly, other participants saw little point in doing things online. Chakkrit argued, “after all, you can go on YouTube and get search results immediately. I wouldn’t even have to wait, like I do for classes. So I didn’t really see a reason for participating [in the online programs]” (Chakkrit, football). Some participants found their sports did not transfer well to the digital setting, as they needed a training partner or proper facilities to practice in: “For taekwondo it's possible, but badminton is totally different. To apply what I learn from videos to real practice is difficult” (Binh, badminton). Thus, many foreign workers continued to be constrained despite the broad transition to online classes and activities, and ended up giving up participating in sports altogether.

#### 5.1.2. Employers’ warnings

*Our boss told us to be careful to not get COVID-19. If one of us got it, then the employees and the plant itself would be in trouble. So, he reminded us many times to not meet any friends or bring them over, and to not to go out. He said that it'd be highly likely we'd get COVID if we went out, so he told us not to and to always wear a mask. (Ram, taekwondo)*

Foreign workers were reminded to be extra careful to avoid getting infected and potentially affecting other staff and the factory negatively. Because foreign workers were cautioned against holding or attending social gatherings, it directly influenced how they could participate in sports and physical activity. In fact, such discourse was pervasive during the pandemic through a range of Korean government campaigns that encouraged people to practice social distancing and refrain from outdoor activity (48). This discourse-practice nexus cautioning against COVID-19 infection can be seen as a technology of domination, which refers to modes of knowledge production in specific practice which “determine the conduct of individuals and submit them to certain ends of domination” (38). In other words, the technology of domination is a means for constraining choices and capacities for the bestowal of approval for individuals' actions, thereby reproducing social constraints that shape the way in which individuals should be involved (32). This technology also occurred when restrictions on outdoor movement were not enforced in practice:

*Our boss said that we could go out but that we had to be careful. So I just didn't go out. I was worried about getting infected because then I would be unable to go to work and could potentially affect my co-workers too. (Chakkrit, football)*

While Chakkrit was allowed to go out, and his employer didn't strictly prohibit outdoor activity, he didn't exercise these freedoms. His actions were constrained due to fears about the consequences if he were to get infected. Taking Chakkrit as an example, foreign workers placed themselves in the division of a particular practice, represented by either the healthy or unhealthy (those infected with COVID-19), and thus worked hard to ensure they did not fall into the latter category.

#### 5.1.3. Hard to find a practice location

*Because all outdoor activities were prohibited, it was really difficult to find a place to play badminton in; we sometimes found empty lots or spaces in industrial areas. Doing outdoor sports or physical activities was strictly banned at that time, so it was really hard to find a proper place to play. (Binh, badminton)*

The last constraint uncovered were the difficulties experienced by foreign workers when they wanted to find a location to exercise in or play, especially for team sports, football and badminton in this context. These types of sports require specialized venues regularly, and particular facilities including football pitches and table tennis or badminton courts for a set period of time. When the government loosened restrictions later on, it became possible to hold sports events and continue team sports activities. However, these policy changes didn't necessarily benefit foreign workers in a tangible way, as noted by one football player.

*You couldn't even play football when you wanted. You first had to book it [the football pitch) and wait for a time slot to open up. But it was really difficult to get a slot even if you paid; I had to wait for a month without any guarantees. That might have been because they didn't want to lend it out to foreigners. (Khaliun, football)*

Khaliun experienced difficulties finding and booking a location for his team to compete, considering they had to share limited facilities with Korean teams. His experience led to conclude that this was a form of discrimination against foreigners. Similarly, Binh also stated that when he wanted to rent one of the badminton courts dominated by Korean clubs, “at first I thought it was (discrimination against foreigners), but it got better as I played with and got to know the Korean players more.” He originally felt that discrimination against foreigners existed, but his perception changed when he proactively approached Korean participants in the same space. Khaliun noted, “I played football well, so I felt like they [the Korean players] liked me,” indicating that his perceptions changed as well in a similar way. He also joined a Korean football club to continue participating in sports, as he found it difficult to book a football pitch for his original football club. After playing alongside Korean players, he discarded his previous notions that local Koreans discriminated against foreigners in the football community.

We can be sure that finding the appropriate sports facilities was a constraint that inhibited foreign workers from continuing on in team sports. Interestingly, when foreign workers inevitably joined Korean clubs and played alongside Korean participants, a pathway for foreign workers to integrate into Korean society was paradoxically created. In this way, one participant shared, “usually we only play amongst ourselves, but if we had a chance to play with Koreans, it’d be great to share and learn from each other” (Oudom, taekwondo).

### 5.2. Enabling constraints

#### 5.2.1. Recognize the importance of “daily” normality

Online programs and activities, alternatives to the more conventional ways of engaging in in-person sports and physical activity during the pandemic, may contribute to helping foreign workers enhance their knowledge and perfection of skills or techniques in their respective sports (49). This was made possible through the training videos provided by the foreign support centers or other resources like YouTube, which foreign workers relied on in the place of in-person training. Nevertheless, all the foreign workers included in this study recognized the importance of in-person sports participation which could not be replaced by purely online activities; and the adoption of new methods during this adjustment period resulted in new feelings of gratitude for having been able to freely take part in outdoor activities in the past.

*Before COVID-19, there was an instructor who came to the center every week to teach us how to play, which I really enjoyed. But then the program stopped during the pandemic, and I had no choice but to learn [badminton] by myself, which was disappointing. It'd be great if the program could start up again. (Binh, badminton)*

As mentioned above, with the exception of taekwondo programs, other sports and physical activity programs were not provided online to the foreign workers in this study. Other support centers in Gyeonggi province also did not provide online programs for sports other than taekwondo. Foreign workers had to practice at home alone, which provided the opportunity for skill development by repetitively practicing the same movements and enhancing skill knowledge. However, they still longed for outdoor sports and activity:

*It was really inconvenient to do it online, all alone. If I had a partner to train with in person, we could check up on each other. But I had to do everything alone, even if I wanted to train with someone else. (Samnang, taekwondo)*

This indicates that the pandemic drove people to give up things they had previously taken for granted, and paradoxically allowed people to recognize the importance of daily “normality,” which had been forgotten about pre-pandemic. Following that, the pandemic created constraints that enabled foreign workers to recognize the importance of normality, which included being able to engage in, and enjoy playing, their favorite sports with *friends outside, in person, at any time*.

#### 5.2.2. Understand Korea and Koreans more

*I joined two football teams. One is the Daemyung team where I play with Korean people, and the other is the Baegot team which consists of my Vietnamese friends … Booking a field was not difficult at all because the Daemyung team president always helped me do it. When he booked something for Daemyung, he would book at the same time for Baegot as well. (Hung, football)*

Hung took on a leadership role in the Vietnamese community in his region, Gimpo, by leading a Vietnamese football club. At the same time, he also joined a Korean football club and played alongside Koreans. Thanks to this dual role which involved the local community, he was in the position of being able to secure football pitches for his Vietnamese team through the help of his Korean team. A shared experience of some study participants was that they seldom encountered barriers finding and securing proper training facilities once they became involved with the Korean sports community. And their involvement in the Korean sports community was a direct result of pandemic-related policies, including social distancing, which severely restricted their ability to operate their clubs and organize games normally.

Ironically, such constraints functioned to enable foreign workers to better approach, understand, and integrate into Korean society. In this vein, one reflected, “I shared food with Korean people, tried to speak Korean, and learned football skills from Korean players” (Ngai, football). In addition, having the chance to play with Koreans helped some of the foreign workers learn Korean sports etiquette, as another participant noted: “the Korean participants have good manners. They bow to us before the game kickoff, and they’re friendly when they approach us to chat after the game. I think it's great” (Chakkrit, football). Of course, mutual interactions take place as well. In this way, the Korean participants can also get to know the foreign workers, giving them the chance to overturn previous assumptions or biases against them and their home countries. With more opportunities to interact and work together, social harmony and integration in South Korea can be improved.

#### 5.2.3. Transformation into active subjects

*I started a [table tennis] club, not with my Indonesian friends but my Korean friends. There's another table tennis club called “Youngnam Club.” We play away games there, or they come to play us. We take turns playing home and away games once a month. (Farrel, table tennis)*

Considering Foucault's (38) technologies of the self, which states that individuals decide to transform themselves to “attain a certain state of happiness” (p. 18), Farrel can be seen to have transformed into a more “active” subject in table tennis participation. Before COVID-19, he had participated in a table tennis program offered by the Hwaseong Center, but all programs were stopped during the pandemic. In order to continue playing, he started a club with his Korean friend. Farrel stated, “I joined a table tennis club near Jangan-myun district. I pay about 60,000 won for it monthly”. As a foreigner, he enjoyed a reduced fee compared to the regular fee of 100,000 won for Koreans, but it was still more costly than free programs offered by the support center. Ngai, involved in a Korean football club, had a similar experience:

*Some days we have to go to Incheon and other days we have to go to Hwaseong, so we have transportation expenses which are covered by our membership fees. If we travel far, I have to pay about 40,000 to 50,000 won in fees. If it's close by, I only pay about 10,000 to 20,000 won. (Ngai, football)*

After the Gimpo Center stopped in-person programs in line with pandemic policies, Ngai joined a Korean football team knowing that he would have to pay more in order to continue playing football. Likewise, two Cambodian interviewees from the Gimpo Center stated that they remained passionate about taekwondo and in order to “not forget, we practiced together and recorded ourselves training to get feedback from the instructor” (Samnang, taekwondo).

Before the pandemic, the foreign support centers had offered a variety of regular programs with benefits such as no membership fees, the provision of equipment and facilities, and foreign worker-focused program schedules. The centers had taken an active role in encouraging foreign workers to participate in sports and physical activity, consequently placing foreign participants in the position of passive objects in sports participation during the pre-pandemic era. Contrastingly, in the pandemic context the foreign workers had to proactively look for ways to continue playing sports due to policy restraints.

#### 5.2.4. Potential for personal reflection

The majority of the foreign workers interviewed shared that they just gave up on doing anything active and “watched TV shows and movies” at home, instead of looking for other sports more suitable for the online setting. Some reasons they shared were, “I’m just not interested [in doing online activities]” or “I just want to play my sports [and not others].” The researchers' in-depth questions about the pandemic constraints can enable interviewees to look back on their choices in reflective, meaningful ways (50). Similarly, the interviews in the study also encouraged the participants to reflect on why they decided it *had* to be their chosen sports and not others, even in spite of constraints that should have pushed them to consider new options to continue to stay active. For example, taekwondo is a sport quite suited for practicing at home alone, even though the study participants preferred to practice in person with others. When Farrel was asked about whether badminton played a special role in helping him stay healthy and maintain his well-being during the pandemic, he made this connection:

*We should exercise to prevent us from getting the virus. If we play table tennis, we can stay healthy and worry less about getting injured. I actually played football before I started table tennis, but I stopped after getting injured. I've enjoyed table tennis since then. (Farrel, table tennis)*

Likewise, when one participant was asked about what helped them continue playing in spite of restrictions and whether they wanted to become an instructor later on, they answered, “if I improve, I’d like to try getting a master's certificate for taekwondo and teach others by running a small club in my home country.” Thus, it can be seen that certain interview questions provided opportunities for the participants to critically reflect upon their choices, particularly regarding why they (1) continued participating in sports despite the pandemic, (2) why they chose their sports (as opposed to others), and (3) how those sports contributed to their health and well-being during the pandemic.

Golob and Giles (50) illustrate how Foucault's understanding of power can be applied as constraints that both inhibit and enable community-based participatory research to identify the empowerment of marginalized people as “active subjects” (p. 360) in the workings of power. Supporting this argument, researchers following Foucault can provide a more comprehensive understanding of the choices of marginalized people by not only examining how their actions are subject to a certain domination, but by also opening a space for a technology of the subject that allows for the participants to self-reflect. That is, researchers following the Foucauldian theory framework can encourage participants to engage with the critical awareness of current limits, discursive constructions, and certain forms of practice in terms of sports participation (51). In this respect, our Foucauldian approach to the interview questions and the participants' answers opened up a potential space that allowed the study participants to think more about their choices, but unfortunately did not reach Foucault's (12) “ethical subject” due to being unable to think critically.

## 6. Concluding remarks

This paper concerned the COVID-19 outbreak in South Korea and how it constrained foreign worker participation in sports and physical activity, particularly focusing on the dualist nature of these constraints as both inhibitors and enablers of action from a Foucauldian perspective. This paper is an effort to look at the positive outcomes stemming from these constraints, an alternative argument to conventional discourses that frame pandemic constraints as being entirely negative. Relaying the authors' personal experiences related to the research theme, this paper attempted to uncover a more productive, active form of individual actions even against a chaotic societal backdrop. While the COVID-19 pandemic is unprecedented and has brought substantial social, economic, and political change to humanity, this paper sought to empirically present how individuals were willing to understand, adjust to, and transform themselves in reaction to these new challenges. This is poignantly encapsulated by the line spoken by Cooper in the film Interstellar (2014), “We will find a way, Professor, we always have.”

There has been little follow-up research on the dual nature of constraints that are both inhibiting and enabling action since Golob and Giles' (9) study. With their approach acting as a starting point for literature focusing on constraints on immigrant participation in leisure, sports, physical education, and recreation in Canada, this paper contributes to the field of study by extending its scope to also touch upon the study of (1) foreign workers, (2) South Korea as a non-Western developed country, and (3) the COVID-19 pandemic. Unfortunately, this paper did not capture the ways in which foreign workers “fully” transformed themselves as “ethical subjects” (12), which Foucault coined as individuals who transform themselves in the technologies of the subject, within the constraints of the pandemic. That is, in the findings, the participants demonstrated the ways in which they became more active subjects in sports participation during the pandemic, and how they came to understand they would be happy with continuing their favorite sports and leading healthy lives through the questions posed by the researchers who follow Foucault's understanding of power and productive features of constraints that are both inhibiting and enabling. Nevertheless, it does not reach the point of full transformation into Foucault's (12) ethical subjects because they did not show self-prioritization about their choices regarding sports participation, in reaction to pandemic constraints. It does not mean that the foreign participants studied did not have the ability to think critically about certain issues, but rather, that there is a need for further ethnographic participant research to follow up on the examination of how foreign workers in sports participation can transform themselves in reaction to a range of constraints in South Korea through self-reflection and self-problematization, similar to Golob and Giles' (50) community-based participatory research on marginalized groups and Giles' (36) ethnographic field research on Indigenous communities in Canada.

## Data Availability

The original contributions presented in the study are included in the article, further inquiries can be directed to the corresponding author.
